# Serum uric acid is related to liver and kidney disease and 12-year mortality risk after myocardial infarction

**DOI:** 10.3389/fendo.2023.1240099

**Published:** 2023-10-11

**Authors:** Luc Heerkens, Anniek C. van Westing, Trudy Voortman, Isabella Kardys, Eric Boersma, Johanna M. Geleijnse

**Affiliations:** ^1^ Division of Human Nutrition and Health, Wageningen University & Research, Wageningen, Netherlands; ^2^ Department of Epidemiology, Erasmus MC, University Medical Center Rotterdam, Rotterdam, Netherlands; ^3^ Department of Cardiology, Erasmus MC, University Medical Center Rotterdam, Rotterdam, Netherlands

**Keywords:** non-alcoholic fatty liver disease, kidney diseases, glomerular filtration rate, uric acid, mortality

## Abstract

**Objective:**

To study the associations of non-alcoholic fatty liver disease (NAFLD), chronic kidney disease (CKD), and serum uric acid (SUA) in patients with post–myocardial infarction (MI) patients, and the relationship of SUA with 12-year mortality risk.

**Methods:**

We included 3,396 patients (60–80 years old, 78% men) of the Alpha Omega Cohort. Multivariable prevalence ratios (PRs) were obtained for the association of NAFLD [fatty liver index (FLI), ≥77 (women) and ≥79 (men)] with CKD [estimated glomerular filtration rate (eGFR), <60 mL/min per 1.73 m^2^]. We calculated sensitivity and specificity of SUA to detect the (combined) presence and absence of NAFLD and CKD. Cause-specific mortality was monitored from enrolment (2002–2006) through December 2018. Hazard ratios (HRs) for all-cause and cardiovascular disease (CVD) mortality in SUA categories were obtained from multivariable Cox models.

**Results:**

Median baseline FLI was 67 (men, 68; women, 64), and mean ± SD eGFR was 81 ± 20 mL/min per 1.73 m^2^ (17% with CKD). Sex-specific FLI was associated with higher CKD prevalence (PR_tertile3 vs. tertile1_, 1.94; 95% confidence interval: 1.57, 2.39). Baseline SUA was 0.36 ± 0.09 mmol/L. With increasing SUA concentrations, specificity for the presence of NAFLD, CKD, or both increased, and sensitivity decreased. During 12 (interquartile range, 9–14) years of follow-up, 1,592 patients died (713 from CVD). HRs ranged from 1.08 (0.88, 1.32) for SUA ≤0.25 mmol/L to 2.13 (1.75, 2.60) for SUA >0.50 mmol/L vs. SUA >0.30–0.35 mmol/L for all-cause mortality. For CVD mortality, HRs ranged from 1.05 (0.77, 1.44) to 2.43 (1.83, 3.25).

**Conclusions:**

NAFLD and CKD were strongly associated, which was reflected by higher SUA concentrations. SUA was a strong predictor of 12-year mortality risk after MI.

## 1 Introduction

Non-alcoholic fatty liver disease (NAFLD) is the most common cause of chronic liver disease worldwide and is regarded as the hepatic manifestation of metabolic dysregulation (1–3). NAFLD is a condition with a wide spectrum of severity, ranging from simple steatosis to steatohepatitis, fibrosis, and cirrhosis. The Fatty Liver Index (FLI) (4) is a scoring system and an easy tool to predict NAFLD in large observational studies. Chronic kidney disease (CKD) has also emerged as a public health issue in the past decades and is a major cause of death (5). Cross-sectional studies link NAFLD, as assessed by the FLI, and CKD in population-based studies (6, 7) and in patients with diabetes (8), which often co-exist with other cardiometabolic alterations, such as insulin resistance, obesity, and elevated blood pressure (1, 9). However, little is known about this association in patients with established cardiovascular diseases (CVDs).

Both NAFLD and CKD have been associated with elevated serum uric acid (SUA) concentrations (10, 11). Studies in healthy populations have demonstrated the prognostic value of SUA for incidence of NAFLD or CKD (12–15). Because previous studies have shown strong associations between NAFLD and CKD (6–8), SUA might also be a marker for the combined presence of both diseases, reflecting an advanced stage of cardiometabolic disorders. SUA was previously associated with a 1.60-fold higher mortality risk in a study of 10,840 Italian patients with post–myocardial infarction (MI) (16). However, little is known about SUA, as potential marker of NAFLD and CKD (combined), in relation to (CVD) mortality after MI.

In Dutch patients with post-MI of the Alpha Omega Cohort, we studied the association between NAFLD and CKD. We then evaluated the diagnostic performance of SUA to detect the (combined) presence and absence of NAFLD and CKD. Finally, we examined whether SUA as diagnostic marker of NAFLD and CKD is associated with long-term mortality risk.

## 2 Materials and methods

### 2.1 Study design and study population

We used data of the Alpha Omega Cohort, consisting of 4,837 drug-treated Dutch patients (aged 60–80 years, 78% men) with a verified history of MI ≤10 years prior to study enrolment. Venous blood samples and data on lifestyle, diet, health, medication use, and anthropometrics were collected at baseline (17, 18). Patients have been continuously monitored for cause-specific mortality. The study was approved by the medical ethics committee of the Haga Hospital (The Hague, The Netherlands). All patients provided verbal and written informed consent for long-term follow-up.

The current analysis excluded patients with missing baseline data on FLI components (n = 207), on alcohol consumption (n = 427), and on eGFR (n = 117). We further excluded patients with heavy alcohol intake (defined as >30 g/day for men and >20 g/day for women, n = 628) and allopurinol users (n = 62), yielding 3,396 patients for all analyses (**Supplemental Figure 1**).

### 2.2 FLI

Baseline FLI was calculated using body mass index (BMI) (kg/m^2^), waist circumference (cm), gamma glutamyl-transferase (GGT, in U/L), and triglycerides (mg/dL). The score is validated in a Caucasian population-based cohort and is calculated as follows (19):


$$\text{FLI }=\;({\;}^{\text{e}0.953*\text{loge }(\text{triglycerides})\;+\;0.139*\text{BMI }+\;0.718*\text{loge }(\text{ggt})\;+\;0.053*\text{waist circumference }-\;15.745})\;/\;(1{+}^{\text{e}0.953*\text{loge }(\text{triglycerides})\;+\;0.139*\text{BMI }+\;0.718*\text{loge }(\text{ggt})\;+\;0.053*\text{waist circumference }-\;15.745})\times 100$$

FLI was categorized into sex-specific tertiles (T1, <49; T2, ≥49–<77; and T3, ≥77 for women; T1, <56; T2, ≥56–<79; and T3, ≥79 for men), and T3 was used as indicator of NAFLD. Weight and height were measured at the patients’ home or hospital by trained research nurses, and BMI was calculated. Waist circumference (cm) was measured at the midpoint between the bottom rib and the top of the hipbone using a non-elastic tape.

Venous blood samples (30 mL) were drawn fasted (≥8 h, 35% of the analytical sample) or non-fasted and were sent to the laboratory by next-working-day mail service at ambient temperatures. Blood samples were immediately processed and stored at −80°C upon arrival (20). Serum triglycerides were determined in multiple batches by standard assays (Roche Diagnostics, cat. no. 1488872) on an automated analyzer (Hitachi 912, Roche Diagnostics) with an inter- and intra-assay coefficient of variation (CV) <10%. Serum GGT was determined in one batch after completion of the cohort by standard assays (Abbott Diagnostics, cat. no. 7D6522) on an automated analyzer (ARCHITECT ci8200, Abbott) with an intra- and inter-assay CV <10%.

### 2.3 eGFR and SUA

A particle-enhanced immunonephelometric assay was used to measure serum cystatin C, and the modified kinetic Jaffé method was used to measure serum creatinine as described in detail elsewhere (21). We used the Chronic Kidney Disease Epidemiology Collaboration (CKD-EPI) equation from 2021 to estimate GFR, which includes both serum creatinine and serum cystatin C (22). CKD was defined as eGFR <60 mL/min per 1.73 m^2^ at baseline. SUA was determined from the same venous blood samples using standard assays (Roche Diagnostics, cat. no. 03P3922) on an automated analyzer (ARCHITECT ci8200, Abbott). Intra- and inter-assay CV was <10%.

### 2.4 Mortality endpoints

Endpoints for the analysis of SUA with mortality risk were all-cause mortality and CVD mortality. Patients were monitored for their vital status from baseline until 31 December 2018 through linkage with municipal registries. Data collection on cause-specific mortality occurred in three phases. During the first 40 months of follow-up (2002–2009), information was obtained from the national mortality registries (Statistics Netherlands, CBS), treating physicians, and close family members. Primary and contributing causes of death were coded by an independent Endpoint Adjudication Committee and described in detail elsewhere (17, 18). After 40 months of follow-up through 2012, mortality data were obtained from CBS for primary and contributing causes of death. From 2013 onward, data on only primary cause of death were obtained from CBS. Treating physicians filled out an additional cause-of-death questionnaire (response rate, 67%), which was coded by study physicians who were not involved in the current analysis. The endpoint CVD or CHD was allocated to all patients for whom it was a primary or contributing cause of death, based on any of the data sources. Fatal endpoints were coded according to the International Classification of Diseases, 10th revision (ICD-10) (23). CVD mortality comprised CHD (codes I20–I25), cardiac arrest (I46), heart failure (I50), stroke (I60–I69), and undefined sudden death (R96). CHD mortality comprised I20–I25, I46, and R96.

### 2.5 Other measurements

At baseline, data on sociodemographic factors and lifestyle habits were collected through self-administered questionnaires as described in detail elsewhere (17). Smoking status was categorized into four categories (current; former, ≤10 years; former, >10 years; and never). Alcohol consumption was assessed with a 203-item validated food frequency questionnaire (24), and ethanol intake (g/day) was computed. Alcohol consumption was then categorized as abstainers (0 g/day), light (>0–10 g/day for men and >0–5 g/day for women), and moderate consumption (>10–30 g/day for men and >5–20 g/day for women). The 2015 Dutch Healthy Diet index (DHD15-index) score was calculated to reflect adherence to the dietary guidelines [scale from no adherence (0) to maximal adherence (150)] (25). Liver enzymes (U/L), alanine aminotransferase and aspartate aminotransferase, were determined by standard assays (Abbott Diagnostics, cat. nos. 8L9222 and 8L9122) on an automated analyzer (ARCHITECT ci8200, Abbott) with an inter- and intra-assay CV <10% from stored blood samples. Blood lipids [mmol/L; total serum cholesterol and high-density lipoprotein cholesterol (HDL-c)] and plasma glucose (mmol/L) were analyzed using standard kits (Hitachi 912, Roche Diagnostics, Basel, Switzerland). The Friedewald formula was used to calculate low-density lipoprotein cholesterol (LDL-c) (26). Patients with BMI ≥30 kg/m^2^ were classified as having obesity. Diabetes mellitus was considered present in case of a self-reported physician’s diagnosis, use of glucose-lowering medication, or elevated plasma glucose (≥7.0 mmol/L if fasted >4 h or ≥11.0 mmol/L if not fasted). Systolic blood pressure (SBP) and diastolic blood pressure (DBP) were measured twice on the left arm with the patient in a seated position, using an automated device (Omron HEM-711) following a 10-min rest. The values were then averaged. Self-reported medication use was checked by trained research nurses and coded according to the Anatomical Therapeutic Chemical Classification System (27): statins (C10AA), antihypertensive drugs (C02, C03, C07, C08, and C09), glucose-lowering drugs (A10), renin-angiotensin-aldosterone system blockers (C09), and diuretics [C03, including thiazides (C03A) and high-ceiling diuretics (C03C)].

### 2.6 Statistical analysis

We visually checked the distribution of the data by using histograms. Baseline characteristics are presented across sex-specific FLI tertiles. Normally distributed variables are presented as means ± standard deviation (SD). Medians and interquartile range (IQR) are used for skewed data, and n (%) is used for categorical data.

We used Cox proportional hazard regression models with follow-up time equal to 1 and robust variances [prevalence ratios (PRs) and 95% confidence intervals (CIs)] to analyze the association between FLI in sex-specific tertiles (T1 as reference) and prevalent CKD.

PRs in the first model were adjusted for age, sex, and fasting state (<8 h, ≥8 h). Model 2 additionally included smoking status (never, former quit ≤10 years ago, former quit >10 years ago, and current), alcohol consumption (abstainers, light, and moderate), statin use (yes or no), and time since last MI. To test the robustness of the results, we repeated analyses subsequently excluding patients with obesity or diabetes. The P_trend_ was obtained by analysing sex-specific FLI tertiles as continuous variable. We also analyzed the association of FLI as a continuous variable with prevalent CKD using restricted cubic splines (RCSs) in men and women separately. Three knots at the 10th, 50th, and 90th percentiles were used according to Akaike’s information criteria, using the median of T1 (30 for women and 41 for men) as the reference. The Wald chi-square test was used to test for non-linearity.

We analyzed the relationship of FLI and eGFR with SUA (all as continuous variables) with RCS and visualized these in three-dimensional plots. We used model 2 of the previously mentioned FLI-CKD analysis, and we additionally added diuretics use (yes or no) and total serum cholesterol. Furthermore, multivariable linear regression models were used to examine the associations of sex-specific FLI tertiles (T1 as reference) and of prevalent CKD (no CKD as reference) with SUA. We used model 2 also including diuretics use (yes or no) and total serum cholesterol and additionally adjusted analyses of FLI for eGFR and vice versa. Analyses were repeated after excluding patients with obesity or diabetes, and thiazides and high-ceiling diuretics use, as these may affect SUA concentrations (28).

We calculated the proportion of patients with the combined absence of NAFLD and CKD, the presence of NAFLD, the presence of CKD, and the combined presence of NAFLD and CKD per interval of 0.05 mmol/L SUA across the range of the 10th to 90th percentile of SUA (0.25–0.50 mmol/L). Sensitivity and specificity analyses were used to assess the diagnostic utility of SUA to detect the four combinations of the (combined) presence and absence of NAFLD and CKD. In prospective analyses, we then used Cox proportional hazard regression models [hazard ratios (HRs) with 95% CIs] to examine the association between SUA and (CVD) mortality risk. SUA was analyzed in intervals of 0.05 mmol/L across the range of the 10th to 90th percentile (0.25–0.50 mmol/L), and we used >0.30–0.35 mmol/L as the reference. Proportional hazard assumptions were examined by log-minus-log survival plots and were met. Survival time was defined as the period between date of baseline assessment and date of death or end of follow-up (for participants who survived), whichever came first. Patients who died due to a competing risk were censored, in addition to those who were lost to follow-up and survived until the end of follow-up. One patient was lost to follow-up and censored after 2.9 years. Models were adjusted for the same variables as in the analyses of FLI, eGFR, and SUA. Analyses were repeated after excluding 748 women, 803 obese patients, 696 patients with diabetes, 805 diuretics users, 1,698 patients with DHD15-index score <80 (below median-split), or 544 current smokers. We also analyzed the association between SUA as continuous variable and (CVD) mortality, using RCS with three knots (10th, 50th, and 90th percentiles) and the median of the group >0.30–0.35 mmol/L as the reference (0.33 mmol/L).

Missing data on fasting state (n = 61) and time since last MI (n = 7) were imputed using multiple imputation with chained equations (with 10 imputations and 10 iterations) using the MICE package (29). The analyses were performed in each imputed dataset separately, and the estimates were subsequently pooled using Rubin’s rules (30).

We used RStudio version 3.6.0 for all analyses, and a two-sided p-value <0.05 was considered statistically significant.

## 3 Results

### 3.1 Baseline characteristics

The patient characteristics across sex-specific FLI tertiles at baseline are presented in **Table 1**. Patients were 69 ± 6 years old and predominantly men (78%). Patients in FLI T3 had a lower eGFR and had higher SUA concentrations than patients in FLI T1. Furthermore, 61% of the patients in FLI T3 were obese, 31% had diabetes, and 97% had hypertension.

**Table 1:** Baseline characteristics of 3,396 patients with post-MI of the Alpha Omega Cohort across sex-specific FLI tertiles.

	Sex-specific FLI		
	T1 M: <56 W: <49 (n = 1,133)	T2 M: ≥56–<79 W: ≥49–<77 (n = 1,131)	T3 M: ≥79 W: ≥77 (n = 1,132)
FLI	39.0 (27.8, 47.6)	67.2 (61.2, 72.9)	88.7 (83.4, 94.1)
Fasting FLI^a^	39.9 (28.7, 47.8)	66.1 (60.4, 72.0)	88.5 (83.2, 94.1)
Sociodemographic factors			
Age, years	69.5 ± 5.4	69.6 ± 5.5	68.5 ± 5.6
Men, n (%)	883 (77.9)	882 (78.0)	883 (78.0)
Lifestyle			
Smoking status, n (%)			
Never	243 (21.4)	219 (19.4)	156 (13.8)
Former, > 10 years	209 (18.4)	206 (18.2)	190 (16.8)
Former, ≤ 10 years	485 (42.8)	541 (47.8)	603 (53.3)
Current	196 (17.3)	165 (14.6)	183 (16.2)
Alcohol consumption^b^, n (%)			
Abstainers	69 (6.1)	68 (6.0)	74 (6.5)
Light	657 (58.0)	685 (60.6)	712 (62.9)
Moderate	407 (35.9)	378 (33.4)	346 (30.6)
DHD15-index score	82.3 ± 13.8	80.2 ± 13.1	78.8 ± 12.7
Kidney function			
eGFR, mL/min per 1.73 m^2^	83.4 ± 19.3	80.6 ± 20.1	78.0 ± 21.6
CKD^c^, n (%)	146 (12.9)	180 (15.9)	242 (21.4)
SUA, mmol/L	0.33 ± 0.08	0.37 ± 0.09	0.39 ± 0.10
Liver enzymes			
Serum GGT, U/L	25.0 (19.0, 32.0)	32.0 (24.0, 44.0)	42.0 (30.0, 63.0)
Fasting serum GGT^a^, U/L	26.0 (20.0, 33.0)	34.0 (25.0, 44.0)	46.0 (32.0, 69.0)
Serum ALT^d^, U/L	15.0 (11.0, 19.0)	16.0 (13.0, 21.0)	19.0 (14.0, 25.3)
Serum AST, U/L	27.0 (23.0, 31.0)	27.0 (23.0, 32.0)	28.0 (24.0, 33.0)
AST/ALT ratio^a^	1.95 ± 0.76	1.78 ± 0.58	1.62 ± 0.94
Cardiovascular (risk) factors			
Time since MI^d^, years	3.05 (1.40, 5.84)	3.53 (1.70, 6.12)	4.15 (1.93, 6.76)
Fasting at blood collection^a^, n (%)	452 (41.5)	418 (38.7)	299 (27.4)
BMI, kg/m^2^	24.6 ± 2.12	27.3 ± 2.03	31.2 ± 3.53
Obesity, n (%)	3 (0.3)	112 (9.9)	688 (60.8)
Waist circumference, cm	92.8 ± 7.28	101 ± 5.88	111 ± 8.49
Serum blood lipids, mmol/L			
Total cholesterol	4.51 ± 0.91	4.63 ± 0.88	4.85 ± 1.02
LDL cholesterol^d^	2.53 ± 0.79	2.57 ± 0.76	2.57 ± 0.90
HDL cholesterol	1.38 ± 0.35	1.24 ± 0.29	1.16 ± 0.29
Triglycerides	1.20 (0.98, 1.55)	1.69 (1.30, 2.18)	2.31 (1.69, 3.12)
Fasting triglycerides^a^	1.12 (0.93, 1.39)	1.56 (1.18, 1.96)	1.94 (1.51, 2.66)
Hypercholesterolemia^e^, n (%)	1079 (95.2)	1087 (96.1)	1095 (96.7)
Plasma glucose^d^, mmol/l	5.63 ± 1.46	6.02 ± 1.90	6.87 ± 2.58
Diabetes, n (%)	139 (12.3)	201 (17.8)	356 (31.4)
SBP^d^, mmHg	140 ± 21.7	142 ± 21.3	142 ± 21.1
DBP^d^, mmHg	78.9 ± 10.7	80.2 ± 11.0	80.9 ± 11.3
Hypertension^f^, n (%)	1052 (92.9)	1078 (95.3)	1099 (97.1)
Medication use, n (%)			
Statins	986 (87.0)	978 (86.5)	957 (84.5)
Antihypertensives	978 (86.3)	1017 (89.9)	1,049 (92.7)
Diuretics	174 (15.4)	259 (22.9)	372 (32.9)
Thiazides	25 (2.2)	49 (4.3)	57 (5.0)
High-ceiling diuretics	119 (10.5)	170 (15.0)	267 (23.6)
RAAS blockers	581 (51.3)	631 (55.8)	694 (61.3)
Diuretics and RAAS blockers	130 (11.5)	187 (16.5)	286 (25.3)

Values are means ± SDs for normally distributed variables, medians (IQRs) for skewed variables, or n (%) for categorical variables.

a Means ± SDs for fasting FLI, fasting triglycerides, and fasting GGT are based on n = 1,169, including only patients who consumed their last meal ≥8 h before blood sampling. Part of the cohort had missing values for fasting state (n = 137).

b Abstinence, 0 g/day; light, >0–10 g/day for men and >0–5 g/day for women; moderate, >10 g/day for men and >5 g/day for women.

c Defined as eGFR <60 mL/min per 1.73 m^2^.

d Part of the cohort had missing values for ALT and AST/ALT ratio (n = 41), time since last MI (n = 34), LDL cholesterol (n = 166), plasma glucose (n = 15), and SBP and DBP (n = 4).

e Defined as use of lipid-lowering medication or total serum cholesterol levels ≥5 mmol/L.

f Defined as use of antihypertensives or either SBP ≥140 mmHg or DBP ≥90 mmHg. MI, myocardial infarction; FLI, fatty liver index; DHD15-index, Dutch Healthy Diet 2015 index; eGFR, estimated glomerular filtration rate; CKD, chronic kidney disease; SUA, serum uric acid; GGT, gamma glutamyl transferase; ALT, alanine aminotransferase; AST, aspartate aminotransferase; BMI, body mass index; LDL-c, low-density lipoprotein cholesterol; HDL-c, high-density lipoprotein cholesterol; SBP, systolic blood pressure; DBP, diastolic blood pressure; RAAS, renin-angiotensin aldosterone system.

### 3.2 Association between FLI and CKD

**Figure 1:**
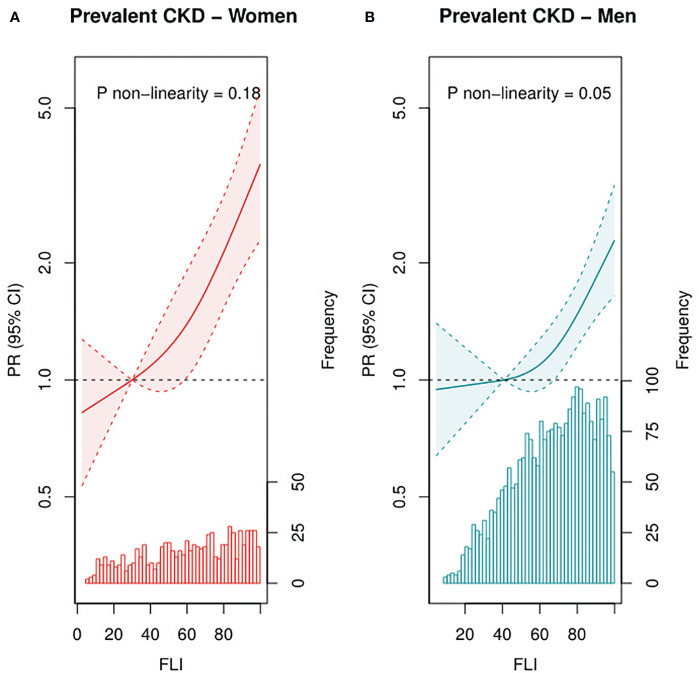
Associations of FLI as continuous variable with prevalent CKD among female (n = 748, **A**) and male (n=2,648, **B**) patients with post-MI of the Alpha Omega Cohort. Solid lines represent PRs, and dashed lines represent 95% CIs. The histogram represents the distribution of FLI. Three-knot restricted cubic splines were used, with the median of T1 (FLI of 30 for women and 41 for men) as reference point. PRs were adjusted for age, fasting state (<8 h, ≥8 h), smoking status (never, former quit ≤10 years ago, former quit >10 years ago, and current), alcohol consumption (abstainers, light, and moderate), time since last MI, and statin use (yes or no). Prevalent CKD defined as eGFR <60 mL/min per 1.73 m^2^. CKD, chronic kidney disease; PR, prevalence ratio; CI, confidence interval; FLI, fatty liver index; MI, myocardial infarction.

At baseline, median (IQR) FLI was 67 (48–83), and mean ± SD eGFR was 81 ± 20 mL/min per 1.73 m^2^ (17% with CKD). In T3 of FLI, 21% had CKD, whereas this was 13% in T1 of FLI. After multivariable adjustment, FLI was associated with higher CKD prevalence, with a PR (95% CI) of 1.94 (1.57, 2.39) for patients in T3 vs. T1 (**Table 2**). This association was confirmed in RCS in strata of men and women (**Figure 1**) and remained strong and statistically significant after excluding patients with obesity or diabetes (**Supplemental Table 1**).

**Table 2:** Associations of sex-specific FLI tertiles with prevalent CKD in 3,396 patients with post-MI of the Alpha Omega Cohort.

	Sex-specific FLI			
	T1 M: <56 W: <49	T2 M: ≥56–<79 W: ≥49–<77	T3 M: ≥79 W: ≥77	P_trend_
Prevalent CKD^a^				
Events/n	146/1133	180/1131	242/1132	
Model 1^b^	REF	1.24 (1.00, 1.54)^d^	1.98 (1.61, 2.44)	<0.001
Model 2^c^	REF	1.24 (0.99, 1.54)	1.94 (1.57, 2.39)	<0.001

a Defined as estimated glomerular filtration rate <60 mL/min per 1.73 m^2^ at baseline.

b Adjusted for sex, age, and fasting state (<8 h, ≥8 h).

c Additionally adjusted for smoking status (never, former quit ≤10 years ago, former quit >10 years ago, and current), alcohol consumption (abstainers, light, and moderate), time since last MI, and statin use (yes or no).

d Prevalence ratio (95% confidence interval) obtained from Cox proportional hazard models, with follow-up time equal to 1, and robust variances (all such values). Abbreviations: FLI, fatty liver index; CKD; chronic kidney disease; MI, myocardial infarction.

### 3.3 Association between FLI, eGFR, and SUA

**Figure 2:**
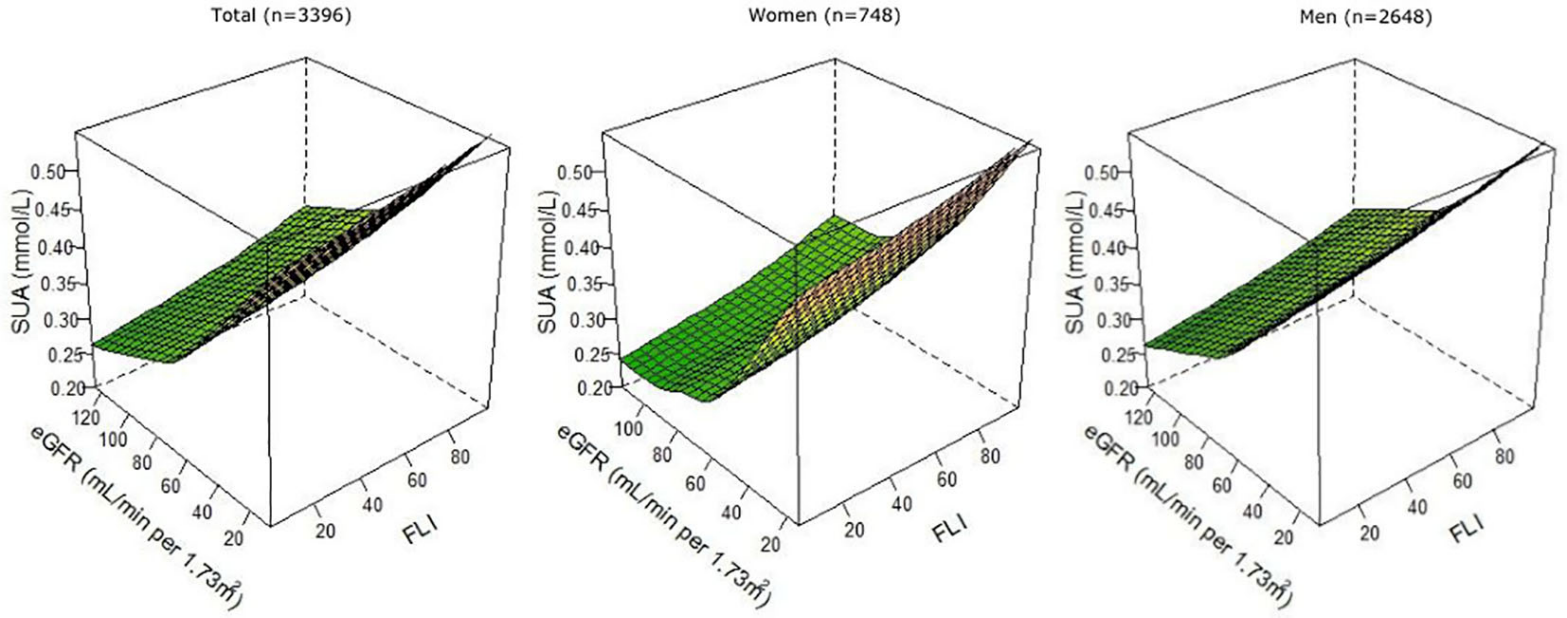
Associations of FLI and eGFR as continuous variables with SUA, overall, and among female (n = 748) and male (n = 2,648) patients with post-MI of the Alpha Omega Cohort. Three-knot restricted cubic splines were used. Values were adjusted for age, sex (except when stratified), fasting state (<8 h, ≥8 h), smoking status (never, former quit ≤10 years ago, former quit >10 years ago, and current), alcohol consumption (abstainers, light, and moderate), time since last MI, statin use (yes or no), total serum cholesterol, and diuretics use (yes or no). FLI, fatty liver index; eGFR, estimated glomerular filtration rate; MI, myocardial infarction; SUA, serum uric acid.

At baseline, the mean ± SD SUA concentration was 0.36 ± 0.09 mmol/L. Patients in T3 of FLI had on average 0.041 mmol/L (95% CI: 0.035, 0.048) higher SUA concentrations than patients in T1, and patients with CKD had on average 0.073 mmol/L (95% CI: 0.065, 0.080) higher SUA concentrations than patients without CKD (**Supplemental Table 2**). Results remained similar after excluding patients with obesity or diabetes (**Supplemental Table 2**). After exclusion of patients using thiazides or high-ceiling diuretics, differences in SUA concentrations were slightly larger, especially for CKD. When FLI, eGFR, and SUA were analyzed as continuous variables in RCS, we observed that patients with the highest FLI and lowest eGFR had the highest SUA concentration, after multivariable adjustment (**Figure 2**).

### 3.4 Association of SUA as diagnostic marker of NAFLD and CKD with mortality

**Figure 3:**
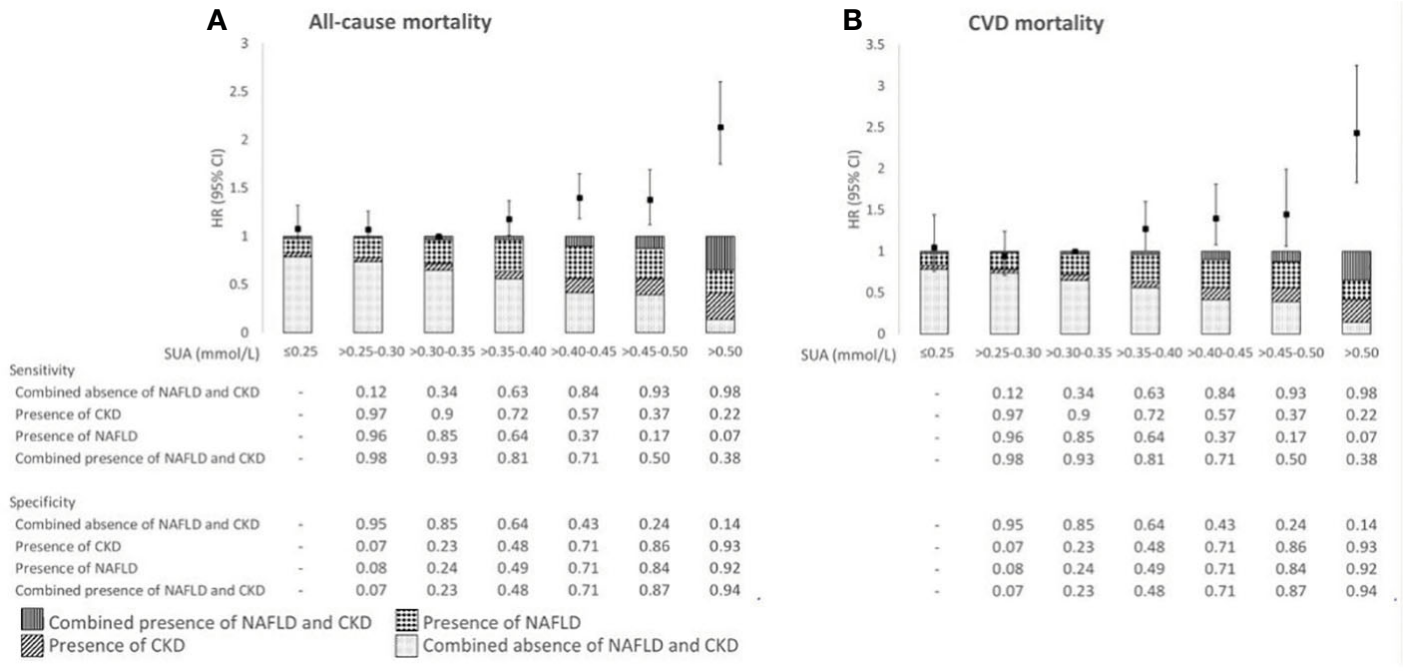
Associations of SUA in relation to all-cause mortality **(A)** and CVD mortality risk **(B)**, proportion of the (combined) absence and presence of NAFLD and CKD, and sensitivity and specificity across categories of SUA in 3,396 patients with post-MI of the Alpha Omega Cohort. HRs were adjusted for age, sex, fasting state, smoking status, alcohol consumption, time since last MI, statin use, total serum cholesterol, and diuretics use. NAFLD, non-alcoholic fatty liver disease; CKD, chronic kidney disease; HR, hazard ratio; CI, confidence interval; SUA, serum uric acid; CVD, cardiovascular disease.

In **Figure 3**, the proportions of patients with the four combinations of (combined) presence and absence of NAFLD and CKD are presented over the range of SUA concentrations. With higher SUA concentrations, proportions of patients with either NAFLD, CKD or both conditions increased, with the latter group being the largest at SUA concentrations >0.50 mmol/L [35% vs. 23% (NAFLD), 28% (CKD), and 14% (none)]. With higher SUA concentrations, the specificity for NAFLD, CKD, or both increased, and the sensitivity decreased. The highest specificity and lowest sensitivity of SUA to detect NAFLD, CKD, or both conditions were reached at SUA concentrations >0.50 mmol/L.

During a median (IQR) follow-up of 12.4 (8.6–13.8) years, 1,592 patients died (713 from CVD). After multivariable adjustment, SUA concentrations ≤0.30 mmol/L were not associated with (CVD) mortality risk (**Figure 3**; **Supplemental Table 3**). However, SUA concentrations >0.35 mmol/L were associated with higher all-cause mortality risk as compared with SUA concentrations >0.30–0.35 mmol/L, with HRs (95% CIs) ranging from 1.18 (1.01, 1.37) for SUA >0.35–0.40 mmol/L through 2.13 (1.75, 2.60) for SUA >0.50 mmol/L. Similarly for CVD mortality, we observed the largest risk estimate for SUA concentrations >0.50 mmol/L [HR 2.43 (95% CI: 1.83, 3.25)] (**Figure 3**; **Supplemental Table 3**). Associations for (CVD) mortality were supported by RCS in which SUA was analyzed as continuous variable (**Supplemental Figure 2**). Results remained largely similar in sensitivity analyses, such as after exclusion of women and patient with obesity or diabetes (**Supplemental Table 4**).

## 4 Discussion

Our findings showed a strong cross-sectional association between NAFLD, as assessed by FLI, and CKD in drug-treated patients with post-MI. Furthermore, FLI and CKD were positively associated with SUA concentrations at baseline. Finally, SUA, as marker of NAFLD and CKD, was associated with a more than two-fold higher (CVD) mortality risk.

To our knowledge, data on the association between FLI and CKD in patients with post-MI are lacking. In a population-based study of 9,436 Chinese adults, higher FLI was associated with higher odds of prevalent CKD, in line with our findings (7). A meta-analysis of cross-sectional population-based studies showed that NAFLD, diagnosed by either ultrasound, histology, or biochemistry, was associated with a two-fold higher odds of prevalent CKD (4). In addition, in our study among patients with post-MI, we found that men and women in the highest vs. lowest tertile of FLI had almost two-fold higher CKD prevalence.

Previous population-based studies have examined the cross-sectional relationship of NAFLD or CKD with SUA but not of both conditions simultaneously. In 5,370 healthy men and women aged 20–74 years of the Third National Health and Nutrition and Examination Survey, participants with hyperuricemia were 40% more likely to have NAFLD (assessed by ultrasound), compared with participants without hyperuricemia (11). Previous Mendelian randomization (MR) studies, using genetic variants to examine causal effects, found no evidence of SUA being a causal risk factor for NAFLD (31, 32). On the contrary, SUA was elevated upon the consequence of NAFLD (31). SUA was positively associated with CKD prevalence in 5,808 elderly of the Cardiovascular Health Study (10). A meta-analysis of randomized controlled trials of patients with CKD showed that SUA-lowering therapy might mitigate the worsening of kidney function (33). However, included trials had a small sample size, there was substantial heterogeneity among trials, and only three of the 12 included studies were double-blinded and placebo-controlled. Therefore, it remains unclear whether SUA plays a causal role in the development of CKD. Nevertheless, SUA, in addition with other markers, has been shown to be a predictor for NAFLD and CKD. Further research is warranted as to whether the addition of SUA might improve the risk assessment of NAFLD and CKD combined.

Previous studies in healthy populations found that prevalent CKD (10) and NAFLD (assessed by ultrasonography) (11) were associated with elevated SUA concentrations. With increasing SUA concentrations, we found larger proportions of patients with either NAFLD, CKD, or both conditions, and this was translated into a higher mortality risk. We observed a more than two-fold higher risk of (CVD) mortality for patients with SUA concentrations >0.50 mmol/L, with patients having both NAFLD and CKD being the largest group. In the GISSI-Prevenzione Trial of 10,840 patients with post-MI and 3.5 years follow-up, patients with SUA concentrations >0.38 vs. <0.25 mmol/L had a 1.60-fold and 1.40-fold higher risk of all-cause and CVD mortality, respectively (16). More than 85% of patients in the Alpha Omega Cohort were treated according to current therapeutic strategies, whereas this was <50% in the GISSI-Prevenzione Trial. Untreated cardiovascular risk factors (i.e., blood pressure) in patients with post-MI may dilute the association of SUA with mortality, which could explain the differences in mortality risk between the Alpha Omega Cohort and GISSI-Prevenzione Trial. However, it remains controversial whether SUA is causal risk factor for CVD. Urate-lowering intervention studies and MR studies failed to show SUA being causally related to CVD events and intermediate risk factors, such as inflammation (34). Therefore, elevated SUA concentrations might merely be a reflection of metabolic dysregulation, such as the presence NAFLD and CKD as shown in the current study.

Strengths of the current study include the large cohort of stable patients with post-MI with detailed data on potential confounders. Limitations include the use of a proxy measure (4) as indicator of NAFLD, whereas imaging techniques are preferred. However, FLI is a validated marker, based on ultrasonography (16), to predict NAFLD and is easier to implement in epidemiological cohort studies. Approximately one-third of our cohort provided fasting blood samples. Non-fasting samples may yield higher serum triglyceride and GGT concentrations. However, results did not change after adjustment for fasting state. Third, kidney function was not measured but estimated. However, eGFR has been widely accepted and used as a valid measure of kidney function in clinical practice (22). Furthermore, using one measurement of SUA may not be reliable. However, within the patients with a repeated measurement of SUA after 40 months of follow-up, the Pearson’s correlation coefficient between two measurements of SUA was 0.71 (95% CI: 0.68, 0.73), indicating a good stability. Finally, because of the observational design of the current study, we cannot prove a causal relationship between NAFLD and CKD or that SUA is causal risk factor of mortality.

To conclude, our results show that NAFLD and CKD are strongly related in patients with post-MI. The strong interrelationship between NAFLD and CKD is, in turn, reflected by elevated SUA concentrations and associated with higher mortality risks. SUA can act as an additional biomarker to improve risk prediction for patients with post-MI, as previously shown in the GISSI-Prevenzione Trial (16). As higher SUA concentration has been associated with multiple cardiometabolic diseases, a diagnostic model based on a single biomarker for NAFLD and CKD is likely unreliable. Therefore, further research is warranted as to whether SUA, with the addition of standard risk markers, can provide a more time- and cost-efficient risk assessment for NAFLD and CKD combined in patients with post-MI.

## Data availability statement

The raw data supporting the conclusions of this article will be made available by the authors upon reasonable request.

## Ethics statement

The studies involving humans were approved by the medical ethics committee of the Haga Hospital (The Hague, the Netherlands). The studies were conducted in accordance with the local legislation and institutional requirements. The participants provided their written informed consent to participate in this study.

## Author contributions

LH, AW, TV, and JMG designed the study; LH and AW analyzed the data and drafted the manuscript; LH, AW, IK, EB, TV, and JMG interpreted the results and revised the manuscript; all authors approved the final version of the manuscript.
