# Supplementary material for: Serum uric acid is related to liver and kidney disease and 12-year mortality risk after myocardial infarction
Source: Front Endocrinol (Lausanne). 2023 Oct 11;14:1240099. doi: 10.3389/fendo.2023.1240099 (PMC10599137; doi:10.3389/fendo.2023.1240099)
Supplement: Supplementary file 1 [file DataSheet_1.docx]

Supplementary Material

Serum uric acid is related to liver and kidney disease and 12-year mortality risk after myocardial infarction

**Luc Heerkens^*^, Anniek C. van Westing, Trudy Voortman, Isabella Kardys, Eric Boersma, Johanna M. Geleijnse**

*** Correspondence:** Luc Heerkens: luc.heerkens@wur.nl

**Supplemental Table 1** Sensitivity analyses for associations of sex-specific FLI tertiles^a^ with prevalent CKD in post-MI patients of the Alpha Omega Cohort.

|  | Sex-specific FLI |  |  |  |
| --- | --- | --- | --- | --- |
|  | T1  M: <56  W: <49 | T2  M: ≥56-<79  W: ≥49-<77 | T3  M: ≥79  W: ≥77 | P_trend_ |
| Prevalent CKD^b^ |  |  |  |  |
| *No obesity (n=2593)* |  |  |  |  |
| Events/n | 145/1130 | 164/1019 | 93/444 |  |
| Model 2^c^ | REF | 1.28 (1.02, 1.60)^d^ | 1.87 (1.43, 2.44) | <0.001 |
| *No diabetes (n=2700)* |  |  |  |  |
| Events/n | 125/994 | 139/930 | 147/776 |  |
| Model 2 | REF | 1.20 (0.94, 1.53) | 1.78 (1.40, 2.27) | <0.001 |

^a^ Sex-specific FLI tertiles are based on n=3396. ^b^ Defined as estimated glomerular filtration rate <60 mL/min per 1.73 m^2^ at baseline. ^c^ Adjusted for sex, age, fasting state, smoking status, alcohol consumption, time since last MI, and statin use. ^d^ Prevalent ratios (95% confidence intervals) obtained from Cox proportional hazards models, with follow-up time equal to 1, and robust variances (all such values), using T1 as the reference. Abbreviations: FLI, Fatty Liver Index; CKD; chronic kidney disease; MI, myocardial infarction.

**Supplemental Table 2** Sensitivity analyses for associations of sex-specific FLI tertiles^a^ and prevalence of CKD with SUA in post-MI patients of the Alpha Omega Cohort.

|  | FLI | | |  | CKD^b^ | |
| --- | --- | --- | --- | --- | --- | --- |
|  | T1  M: <56  W: <49 | T2  M: ≥56-<79  W: ≥49-<77 | T3  M: ≥79  W: ≥77 |  | No | Yes |
| SUA (mmol/L) |  |  |  |  |  |  |
| *Total* |  |  |  |  |  |  |
| N | 1133 | 1132 | 1132 |  | 2828 | 568 |
| Mean ± SD | 0.33 ± 0.08 | 0.37 ± 0.09 | 0.39 ± 0.10 |  | 0.35 ± 0.08 | 0.44 ± 0.12 |
| Model 1^c^ | REF | 0.033 (0.026, 0.041)^e^ | 0.064 (0.057,0.072) |  | REF | 0.098 (0.090,0.106) |
| Model 2^d^ | REF | 0.024 (0.018,0.031) | 0.041 (0.035,0.048) |  | REF | 0.073 (0.065,0.080) |
| *No obesity (n=2593)* |  |  |  |  |  |  |
| N | 1130 | 1019 | 444 |  | 2191 | 402 |
| Mean ± SD | 0.33 ± 0.08 | 0.37 ± 0.09 | 0.40 ± 0.10 |  | 0.34 ± 0.08 | 0.43 ± 0.12 |
| Model 2 | REF | 0.024 (0.017,0.030) | 0.043 (0.034,0.051) |  | REF | 0.074 (0.065,0.082) |
| *No diabetes (n=2700)* |  |  |  |  |  |  |
| N | 994 | 930 | 776 |  | 2289 | 411 |
| Mean ± SD | 0.33 ± 0.08 | 0.37 ± 0.09 | 0.39 ± 0.09 |  | 0.35 ± 0.08 | 0.43 ± 0.12 |
| Model 2 | REF | 0.027 (0.021,0.034) | 0.044 (0.037,0.051) |  | REF | 0.068 (0.060,0.076) |
| *No thiazide users (n=3265)* |  |  |  |  |  |  |
| N | 1108 | 1082 | 1075 |  | 2728 | 537 |
| Mean ± SD | 0.33 ± 0.08 | 0.36 ± 0.09 | 0.39 ± 0.10 |  | 0.35 ± 0.08 | 0.44 ± 0.12 |
| Model 2 | REF | 0.027 (0.020,0.033) | 0.048 (0.042,0.055) |  | REF | 0.090 (0.083,0.098) |
| *No high-ceiling users (n=2840)* |  |  |  |  |  |  |
| N | 1014 | 961 | 865 |  | 2489 | 351 |
| Mean ± SD | 0.32 ± 0.07 | 0.35 ± 0.07 | 0.38 ± 0.08 |  | 0.34 ± 0.07 | 0.40 ± 0.10 |
| Model 2 | REF | 0.026 (0.020,0.032) | 0.045 (0.038,0.051) |  | REF | 0.060 (0.052,0.068) |

^a^ Sex-specific FLI tertiles are based on n=3396. ^b^ Defined as eGFR <60 mL/min per 1.73 m^2^ at baseline. ^c^ Adjusted for sex, age, and fasting state. ^d^ Additionally adjusted for smoking status, alcohol consumption, time since last MI, statin use, diuretics use (but not when thiazide or high-ceiling diuretics users were excluded), total serum cholesterol, and FLI (for analyses of eGFR with SUA) or eGFR (for analyses of FLI with SUA). ^e^ β (95% confidence intervals) obtained from multivariable linear models (all such values), using T1 of FLI or no CKD as reference. Abbreviations: FLI, Fatty Liver Index; eGFR, estimated glomerular filtration rate; SUA, serum uric acid; CKD, chronic kidney disease; SD, standard deviation; MI, myocardial infarction.

**Supplemental Table 3** Associations of baseline SUA with 12-year risk of all-cause and CVD mortality in 3396 post-MI patients of the Alpha Omega Cohort.

|  | SUA (mmol/L) | | | | | | |  |
| --- | --- | --- | --- | --- | --- | --- | --- | --- |
|  | ≤0.25 | >0.25-0.30 | >0.30-0.35 | >0.35-0.40 | >0.40-0.45 | >0.45-0.50 | >0.50 | P_trend_ |
| **All-cause mortality** |  |  |  |  |  |  |  |  |
| Events/n | 136/307 | 236/565 | 344/871 | 324/716 | 247/459 | 132/235 | 173/243 |  |
| Person-years | 3539 | 6551 | 10 179 | 8067 | 4854 | 2474 | 1958 |  |
| Model 1^a^ | 1.08 (0.88, 1.31)^c^ | 1.06 (0.90, 1.26) | REF | 1.19 (1.02, 1.38) | 1.46 (1.24, 1.73) | 1.59 (1.30, 1.94) | 2.75 (2.29, 3.30) | <0.001 |
| Model 2^b^ | 1.08 (0.88, 1.32) | 1.07 (0.90, 1.26) | REF | 1.18 (1.01, 1.37) | 1.40 (1.18, 1.65) | 1.38 (1.12, 1.69) | 2.13 (1.75, 2.60) | <0.001 |
|  |  |  |  |  |  |  |  |  |
| **CVD mortality** |  |  |  |  |  |  |  |  |
| Events/n | 56/307 | 91/565 | 148/871 | 152/716 | 108/459 | 62/235 | 96/243 |  |
| Person-years | 3539 | 6551 | 10 179 | 8067 | 4854 | 2474 | 1958 |  |
| Model 1 | 1.01 (0.73, 1.39) | 0.95 (0.73, 1.24) | REF | 1.29 (1.02, 1.62) | 1.50 (1.17, 1.95) | 1.81 (1.34, 2.44) | 3.54 (2.71, 4.61) | <0.001 |
| Model 2 | 1.05 (0.76, 1.44) | 0.94 (0.72, 1.24) | REF | 1.26 (1.00, 1.60) | 1.40 (1.08, 1.81) | 1.45 (1.06, 1.99) | 2.43 (1.83, 3.25) | <0.001 |

^a^ Adjusted for sex, age, and fasting state (<8 hours, ≥8 hours). ^b^ Additionally adjusted for smoking status (never, former quit ≤10 y ago, former quit >10 y ago, current), alcohol consumption (abstainers, light, moderate), time since last MI, statin use (yes, no), total serum cholesterol, and diuretics use (yes, no). ^c^ Hazard ratios (95% confidence intervals) obtained from Cox proportional hazards models (all such values), using SUA >0.30-0.35 mmol/L as the reference. Abbreviations: SUA, serum uric acid; MI, myocardial infarction; CVD, cardiovascular disease.

**Supplemental Table 4** Sensitivity analyses for baseline categories of SUA and risk of all-cause and CVD mortality in post-MI patients of the Alpha Omega Cohort.

|  | SUA (mmol/L) | | | | | | |
| --- | --- | --- | --- | --- | --- | --- | --- |
|  | ≤0.25 | >0.25-0.30 | >0.30-0.35 | >0.35-0.40 | >0.40-0.45 | >0.45-0.50 | >0.50 |
| **All-cause mortality** |  |  |  |  |  |  |  |
| Men (n=2648) | 1.10 (0.85,1.42)^a^ | 1.18 (0.97,1.42) | REF | 1.24 (1.04,1.47) | 1.39 (1.15,1.67) | 1.42 (1.13,1.79) | 2.04 (1.63,2.56) |
| No obesity (n=2593) | 1.13 (0.91,1.40) | 1.08 (0.90,1.30) | REF | 1.22 (1.02,1.46) | 1.46 (1.21,1.77) | 1.41 (1.11,1.79) | 2.14 (1.69,2.72) |
| No diabetes (n=2700) | 1.05 (0.83,1.33) | 1.04 (0.86,1.25) | REF | 1.16 (0.98,1.38) | 1.43 (1.18,1.72) | 1.46 (1.15,1.86) | 2.06 (1.63,2.61) |
| No diuretics (n=2591) | 1.03 (0.83,1.29) | 1.12 (0.94,1.35) | REF | 1.14 (0.96,1.37) | 1.37 (1.12,1.67) | 1.44 (1.09,1.90) | 2.48 (1.75,3.51) |
| High DHD15-index (n=1698) | 0.95 (0.71,1.27) | 0.99 (0.78,1.26) | REF | 1.10 (0.88,1.37) | 1.15 (0.90,1.46) | 1.35 (1.00,1.81) | 1.95 (1.47,2.58) |
| No current smokers (n=2852) | 1.21 (0.96,1.53) | 1.17 (0.97,1.42) | REF | 1.13 (0.95,1.34) | 1.37 (1.14,1.65) | 1.47 (1.17,1.84) | 2.11 (1.69,2.62) |
|  |  |  |  |  |  |  |  |
| **CVD mortality** |  |  |  |  |  |  |  |
| Men (n=2648) | 1.23 (0.82, 1.85) | 1.16 (0.86, 1.85) | REF | 1.44 (1.10, 1.88) | 1.39 (1.00, 1.94) | 1.76 (1.23, 2.50) | 2.59 (1.84, 3.65) |
| No obesity (n=2593) | 1.17 (0.80, 1.71) | 0.97 (0.59, 1.20) | REF | 1.36 (1.01, 1.82) | 1.37 (0.99, 1.91) | 1.58 (1.10, 2.27) | 2.49 (1.77, 3.52) |
| No diabetes (n=2700) | 1.12 (0.78, 1.60) | 0.97 (0.71, 1.32) | REF | 1.34 (1.02, 1.76) | 1.44 (1.06, 1.94) | 1.70 (1.16, 2.48) | 2.51 (1.78, 3.54) |
| No diuretics (n=2591) | 1.01 (0.68, 1.49) | 0.97 (0.71, 1.32) | REF | 1.29 (0.95, 1.74) | 1.30 (0.93, 1.83) | 1.85 (1.09, 2.73) | 3.16 (1.81, 5.50) |
| High DHD15-index (n=1698) | 1.09 (0.65, 1.83) | 0.93 (0.60, 1.46) | REF | 1.57 (1.08, 2.28) | 1.14 (0.74, 1.77) | 1.81 (1.21, 2.83) | 3.19 (1.89, 5.37) |
| No current smokers (n=2852) | 1.19 (0.82, 1.75) | 0.96 (0.70, 1.17) | REF | 1.21 (0.93, 1.56) | 1.30 (0.97, 1.73) | 1.57 (1.12, 2.20) | 2.56 (1.88, 3.50) |

^a^ HR (95% confidence intervals) obtained from Cox proportional hazards models (all such values), using SUA >0.30-0.35 mmol/L as the reference. HRs are adjusted for sex (but not when stratified), age, fasting state, smoking status (but not when stratified), alcohol consumption, time since last MI, statin use, total serum cholesterol, and diuretics use (but not when stratified). Abbreviations: SUA, serum uric acid; MI, myocardial infarction; HR, hazard ratio; CVD, cardiovascular disease; DHD15-index, Dutch Healthy Diet 2015 index.

Alpha Omega Cohort:

n=4837

Patients with complete data on FLI components, alcohol, and eGFR:

n=4086

Analytical sample:

n=3396

Excluded:

N=628 heavy alcohol users

N=62 allopurinol users

Excluded:

N=207 with missing data on baseline FLI components

N=427 with missing data on baseline alcohol consumption

N=117 with missing data on baseline eGFR

**Supplemental Figure 1** **Flow chart for selection of the analytical sample of the Alpha Omega Cohort.** Heavy alcohol consumption is defined as >20 g/day for women and >30 g/day for men. Abbreviations: FLI, fatty liver index; eGFR estimated glomerular filtration rate.


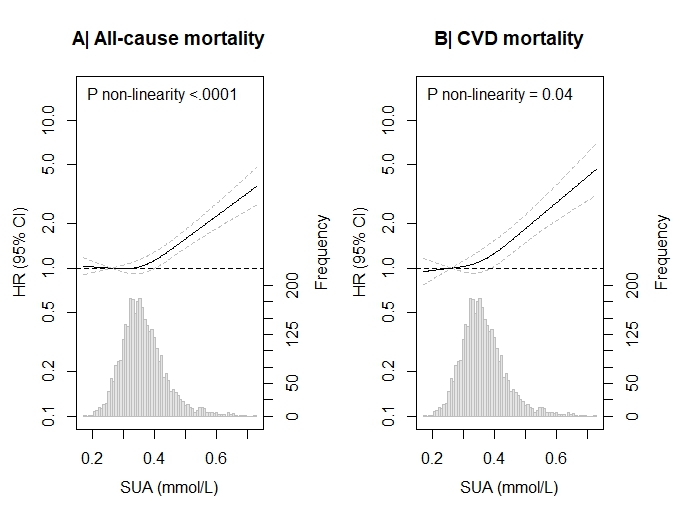


**Supplemental Figure 2** **Associations of SUA as continuous variable with risk of all-cause (A) and CVD mortality (B) among 3396 post-MI patients of the Alpha Omega Cohort.** Solid lines represent HRs and dashed lines represent 95% CIs. The histogram represents the distribution of SUA. Three-knot restricted cubic splines was used, with the median SUA level of the middle SUA category (i.e. >0.30-0.35) as reference point (0.33). HRs were adjusted for age, sex, fasting state, smoking status, alcohol consumption, time since last MI, statin use, total serum cholesterol, and diuretics use. Abbreviations: CVD, cardiovascular disease; HR, hazard ratio; CI, confidence interval; SUA, serum uric acid; MI, myocardial infarction.
